# Chronic Inflammation Offers Hints About Viable Therapeutic Targets for Preeclampsia and Potentially Related Offspring Sequelae

**DOI:** 10.3390/ijms252312999

**Published:** 2024-12-03

**Authors:** Jaya Prasad, Juliette Van Steenwinckel, Alistair J. Gunn, Laura Bennet, Steven J. Korzeniewski, Pierre Gressens, Justin M. Dean

**Affiliations:** 1 Department of Physiology, Faculty of Medical and Health Sciences, University of Auckland, Auckland 1142, New Zealand; j.prasad@auckland.ac.nz (J.P.); aj.gunn@auckland.ac.nz (A.J.G.); l.bennet@auckland.ac.nz (L.B.); j.dean@auckland.ac.nz (J.M.D.); 2 Inserm, Neurodiderot, Université de Paris, 75019 Paris, France; juliette.van-steenwinckel@inserm.fr; 3 C.S. Mott Center for Human Growth and Development, Department of Emergency Medicine, Wayne State University School of Medicine, Detroit, MI 48202, USA; 4 Centre for the Developing Brain, Division of Imaging Sciences and Department of Biomedical Engineering, King’s College London, King’s Health Partners, St. Thomas’ Hospital, London SE1 7EH, UK

**Keywords:** intrauterine growth restriction, small for gestational age, chorioamnionitis, neurodevelopmental, disability, autism, cerebral palsy, hypertension, social vulnerability

## Abstract

The combination of hypertension with systemic inflammation during pregnancy is a hallmark of preeclampsia, but both processes also convey dynamic information about its antecedents and correlates (e.g., fetal growth restriction) and potentially related offspring sequelae. Causal inferences are further complicated by the increasingly frequent overlap of preeclampsia, fetal growth restriction, and multiple indicators of acute and chronic inflammation, with decreased gestational length and its correlates (e.g., social vulnerability). This complexity prompted our group to summarize information from mechanistic studies, integrated with key clinical evidence, to discuss the possibility that sustained or intermittent systemic inflammation-related phenomena offer hints about viable therapeutic targets, not only for the prevention of preeclampsia, but also the neurobehavioral and other developmental deficits that appear to be overrepresented in surviving offspring. Importantly, we feel that carefully designed hypothesis-driven observational studies are necessary if we are to translate the mechanistic evidence into child health benefits, namely because multiple pregnancy disorders might contribute to heightened risks of neuroinflammation, arrested brain development, or dysconnectivity in survivors who exhibit developmental problems later in life.

## 1. Introduction

Preeclampsia (PE) is a hypertensive disorder long characterized as an excessive maternal inflammatory response to pregnancy [1,2] that is associated with increased risk of damage to developing brain structure or function in offspring (even at term) [3]. However, the significance and directions of the associations are inconsistent across clinical studies [4,5]. Causal inferences about risk factors and sequelae are often complicated by an increasingly frequent overlap of PE, fetal growth restriction (FGR), and a milieu of inflammation-related phenomena, with decreased gestational length and its correlates (e.g., social vulnerability) [6], namely because Mill’s method of comparing agreement and differences across samples can be unreliable when multifactorial effects (i.e., mechanisms) involving reinforcement or cancellation are at play [7]. Pre-clinical research helps shed light on etiology in such scenarios, and its integration with observational evidence is critical for translating the insights into therapeutics for human health benefits [8].

We therefore review mechanistic studies and integrate key clinical evidence to examine the general hypothesis that chronic inflammation during critical periods of development conveys information about viable therapeutic targets, not only for the prevention of PE and its correlates (FGR and preterm delivery) but also the neurobehavioral (and other) sequelae that appear to be overrepresented in offspring survivors. First, we explain our focus on chronic inflammation in PE. Next, how systemic inflammation might influence the developing central nervous system (CNS) is discussed, before we introduce the key cellular and molecular players. The remaining sections summarize the most frequently used animal models of neuroinflammation, identify key clinical indicators of perinatal brain injury or dysfunction, and discuss study design implications for the identification of viable therapeutic targets.

**Figure 1:**
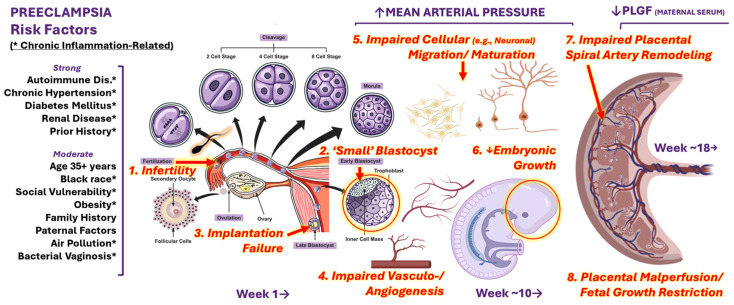
Conceptual Model Linking Preeclampsia Risk Factors, Chronic Inflammation and Potentially Related Sequelae in Offspring. [Chronic inflammation related risk factors for PE (*) might impact fertility or otherwise limit growth/maturation in ways that affect implantation (red numbers 1–3) or distrupt typical developmental processes (red numbers 4–6) that can increase the risk of downstream placental dysfunction (red numbers 7–8) and related clinical diagnoses after the 20th week of gestation. The conceptus is believed to be supported by histotrophic nutrition in early gestation before developing its placenta [11,12,13,14,15,16,17,18,19,20,21,22,23,24]. Around 7–12 weeks, placentation occurs by way of a complex network of interactions between uterine, decidual, and fetal cell populations [25,26,27,28,29,30,31,32]. Disruption of these developmental processes appears to limit trophoblast invasiveness, particularly before the 12th week of pregnancy when markers of trophoblast stemness decline rapidly [33], resulting in shallow placentation and inadequate remodeling of the spiral arteries [34,35,36,37,38,39,40,41], thereby reducing the flow of nutrients in maternal blood to the intervillous space [34,35,36,37,38,39,40,41], where oxygen and nutrients are transported to the fetus [42,43,44,45]. This, in turn, seems to raise the risk of downstream pregnancy and offspring developmental disorders that are associated with sustained systemic inflammation and white matter damage or dysfunction (e.g., PE, FGR, and autism). Thus, early gestation (or even prior to that) might present new opportunities to develop therapeutic interventions not only for PE, but also for optimizing fetal growth and the prevention of related neurobehavioral (and possibly cardiovascular and pulmonary) deficits in offspring [46]. (The ‘Week 1’ portion of the figure was adapted nder an extended license agreement [#505384740] from an Adobe Stock image created by udaix, while the images to the right were created by the authors using BioRender under an Academic License.)].

Figure 1 is an oversimplified but helpful diagram introducing the multiple opportunities for chronic systemic inflammation to affect the developing brain and placental structures or later functioning in the context of preeclampsia and its risk factors [9]. Namely, the chronic inflammation-related phenomena that can impact sperm or otherwise affect fertility (e.g., autoimmune disorders, obesity, air pollution) might also influence cell proliferation, embryo implantation and growth, vasculogenesis/angiogenesis, cellular (e.g., neuronal) migration and/or maturation, uterine spiral artery remodeling, the yolk sac or downstream placental structures or functions that regulate fetal growth or protect the developing brain, heart, and lungs later in gestation. For example, obesity and neuroinflammation appear to convey information about sex-specific reproductive health outcomes [10] that are in turn associated with heightened PE risk.

### 1.1. Why Focus on Preeclampsia?

PE affects 2% to 8% of pregnant women, but accounts for 8% to 27% of maternal deaths worldwide [47]. In the US, the short-term economic costs of PE annually amount to ~USD 2 billion [48]. Long-term costs are much higher because survivors are at increased risk of metabolic syndrome and cardiovascular disease, depression, and cognitive impairment later in life [49,50], as are their offspring [51,52,53,54]. PE is a leading indication for iatrogenic preterm delivery (PTD), but it is also associated with heightened risk of spontaneous PTD, and surviving offspring are at heightened risk of autism spectrum disorders (ASDs) [55,56,57], cerebral palsy [58,59,60,61], and other neurodevelopmental deficits [62] that annually cost the US an estimated USD 85–USD 167 billion [62].

### 1.2. Why Chronic Systemic Inflammation?

Chronic inflammation occurs before, during, and after pregnancy, is strongly associated with hypertension and its correlates both in and outside of pregnancy (e.g., social vulnerability), and also conveys information about increased risks of multiple neurobehavioral and cardiometabolic disorders in mothers and offspring alike [3]. The bulk of observational evidence linking chronic systemic inflammation to perinatal morbidity, however, comes from studies of high-risk pregnancies that typically end early.

The premise for studying high-risk pregnancies that deliver much earlier than expected is that we might find clues about pathophysiology occurring downstream in utero. Major pregnancy complications that are implicated in the pathogenesis of damage to developing brain structure and function among children born extremely preterm can be divided into two clusters based on indicators of underlying biology: acute inflammation-related complications (i.e., preterm labor, preterm premature membrane rupture, placental abruption, and cervical insufficiency), and those that appear more so rooted in malplacentation and chronic inflammation-related phenomena (i.e., PE and delivery for fetal indications/intrauterine growth restriction) [63]. Complexities emerge, however, because of the developmental regulation of what appear to be overlapping risk profiles for both clusters [64]; i.e., indicators of acute and chronic inflammation-related phenomena convey dynamic information about maternal, fetal, and postnatal complications, possibly acting akin to a multi-hit model of neonatal white matter injury [65].

Multiple indicators of maternal immune activation even before pregnancy are associated with increased risk of PE, including but not limited to infections (e.g., cervical–vaginal infections [66,67]) [68]. In the US, 20% to 40% of reproductive-age women have low-grade systemic inflammation (high-sensitive C-reactive protein [hsCRP] ≥ 2 mg/L) [69], and early pregnancy exposure is associated with increased risk of hypertensive pregnancy disorders [70], including PE [71], both in obese and lean women. Indeed, observational studies have shown that maternal blood levels of inflammation-related proteins tend to be dysregulated during the first and second trimesters of pregnancies that later develop hypertension [72] and PE [73,74,75,76]. Women who develop PE often exhibit a mostly pro-inflammatory T helper (Th) 1 immune profile (e.g., heightened interferon [IFN]-γ, interleukin [IL]-6, tumor necrosis factor [TNF]-α, and IL-1β) [72,77,78], whereas unaffected pregnancies shift towards a mostly anti-inflammatory TH2 immune profile (e.g., increased levels of IL-4, IL-10, and IL-13) [79,80]. Importantly, observational evidence supports the possibility of links between low grade systemic inflammation, PE [71], and autism in offspring [81].

### 1.3. What Is Known About Therapeutic Prevention?

Experimental studies demonstrate that early administration of low-dose aspirin (LDA) can prevent PE in some high-risk women [82,83,84], possibly FGR and PTD, too [85], but the underlying mechanism is not yet established [86], and there are lingering questions about optimal dosage [87], eligibility criteria [88], timing [89], and discontinuation criteria [90]. Nor do we know whether similar strategies might offer neuroprotective or other benefits for offspring. We were able to identify just two trials that were conducted in the 1990s [91]; one reported that children exposed antenatally to 50–60 mg aspirin had reduced post-neonatal mortality at one year versus placebo or no treatment [92], while the other trial found fewer motor problems at 18 months among children exposed to antenatal aspirin [93]. A more recent long-term follow-up study of the EAGeR trial of women aged 18–50 years who were attempting to become pregnant after one to two prior losses showed no association between LDA administration and maternal cardiometabolic or offspring respiratory morbidity [94]. How might systemic inflammation affect the developing CNS?

## 2. Crosstalk Between Systemic Inflammation and the CNS

A dynamic crosstalk involving systemic immunity (e.g., circulating immune cells), the endocrine system, and the gut microbiome is thought to play an important role in maintaining CNS homeostasis during development and into adulthood. Although the mechanisms by which the peripheral immune responses to perinatal systemic infection/inflammation propagate to cause central inflammation and brain injury are complex [95,96], there is increasing evidence for dysregulation of these physiological processes.

### 2.1. CNS and Infiltrating Immune Cells

The CNS is an immune-privileged site that is relatively isolated from the peripheral immune system by the blood–brain barrier (BBB). The endothelial cells of the BBB selectively regulate the entry of molecules into the brain tissue and closely interact with neurons, pericytes, and astrocytes to maintain the brains’ immune autonomy [97,98]. Under physiological conditions, there is some limited entry of circulating immune cells and cytokines into the CNS via interactions with antigen-presenting cells, pattern recognition receptors (PRRs, e.g., toll-like receptors [TLRs]), and resident immune cells present in the choroid plexus, meninges, circumventricular organs, and ventricles [99,100]. However, following systemic immune stimulation, there is a rapid systemic inflammatory response mediated by activation of PRRs. PRRs recognize pathogen and damage associated molecular patterns (PAMPs and DAMPs, respectively) released by various microorganisms or from the sites of tissue damage [101]. Activation of PRRs triggers a range of specific intracellular signaling cascades including nuclear factor-κβ signaling. In turn, this increases the production of circulating pro- and anti-inflammatory cytokines (e.g., IL-1β, IL-6, TNF-α, and INF-γ), and other immune-related molecules (e.g., C-reactive protein and prostaglandins), as well as the recruitment of leukocytes [99].

The systemic inflammatory response described above is thought to induce local CNS inflammation via at least three mechanisms, as follows: (1) Disruption of the BBB and increased permeability to immune cells via recruitment and activation of molecules such as matrix metalloproteinases (MMPs), which degrade tight junctions and other extracellular matrix components of the BBB [102]. This increased BBB permeability allows pathogens and peripheral immune cells to infiltrate the brain directly, which can facilitate further entry of inflammatory cells [103,104]. (2) CNS infiltration of cytokines via specific cytokine transporters at the BBB [105]. (3) CNS entry of immune molecules such as peptides, cytokines, and bacterial products in areas devoid of the BBB (e.g., circumventricular organs and the choroid plexus) [106]. Once infiltrating immune cells and cytokines enter the CNS, they can directly propagate the inflammatory response by activating microglia and astrocytes, leading to neural injury (see Section 5) [104,107]. Interestingly, experimental evidence suggests disruption of the BBB in PE [108,109,110,111,112].

### 2.2. Neuro–Endocrine–Immune Axis

Multidirectional communication between the endocrine system, immune system, and the CNS has a key role in maintaining brain homeostasis. However, disturbances in the endocrine system, such as with prenatal stress, can lead to abnormal immune activation or immunosuppressive effects, which can be detrimental to the developing brain [113]. For example, psychosocial stress before and in early pregnancy is associated with increased risk of PE and its correlates [114,115,116,117]. Prenatal stress during the second trimester of pregnancy is also associated with increased concentrations of IL-1β, IL-4, IL-5, IL-6, and IL-8 in newborn infants [118], as is PE, and may contribute to increased risk of adverse developmental outcomes (e.g., preterm delivery, low birth weight, FGR, and cognitive deficits). Both PE [119,120,121] and prenatal depression [122,123,124,125] have been associated with reduced brain derived neurotrophic factor expression in maternal blood, placenta, and umbilical cord blood, which is associated with neurodevelopmental or behavioral problems [126,127,128,129] (although not all studies agree [130]). Reduced brain-derived neurotrophic factor in the amniotic fluid [131] and newborn blood [132] has also been reported in FGR. Therefore, it is possible that neurotrophic factors may convey information about the pathogenesis of neurodevelopmental outcomes in offspring [133,134,135,136]. It is also possible that glucocorticoid release in response to stress can have potent immunosuppressive actions, including reduced cytokine production and inflammation [137,138], which can impact neural function. Indeed, administration of synthetic glucocorticoids in preterm born infants is associated with increased risk of poor neurodevelopmental outcomes, including cerebral palsy and developmental delays [139,140].

Systemic infection/inflammation in preterm born infants is associated with changes in blood levels of thyroid-stimulating hormone (TSH) and insulin-like growth factor (IGF)-1, which play key roles in normal brain development and function [141,142]. For example, reduced TSH concentrations in combination with intermittent or sustained systemic inflammation are associated with significantly increased risk of brain injury and adverse motor and cognitive outcomes in preterm infants [143]. Postnatal inflammation in preterm born infants is also associated with reductions in lower circulating levels of IGF-1, IGF binding protein (BP)-1, and IGFBP-3 compared with healthy term counterparts [144]. This reduction in circulating IGF-1 concentrations is associated with elevated pro-inflammatory cytokine levels (TNF-α and IL-6) [145] and an increased risk of major neonatal morbidities, including poor brain development, decreased total brain volume, and adverse neurodevelopmental outcomes, at 2 years of age [146,147].

### 2.3. Gut–Brain Axis

An emerging concept in the field of organ crosstalk involves the influence of the gut microbiota on brain function and stress responses via a bidirectional network of brain–gut communication. This crosstalk integrates neural, endocrine, and immunological signaling between the gut and brain, and is largely thought to be driven by the intestinal microbiota [148,149]. For example, in germ-free mice, the complete absence of gut microbiota caused an exaggerated hypothalamic–pituitary–adrenal axis response, which was reversed by postnatal microbial colonization [150]. Further, germ-free mice exhibited increased expression of synaptic-related proteins, increased motor activity, and reduced anxiety [151], increased serotonin metabolism in the hippocampus [152], and hypermyelination in the prefrontal cortex [153]. These studies suggest a key role of intestinal microbiota in normal brain development.

During normal development, the gut is rapidly colonized with bacteria around the time of birth [153,154,155,156]. These bacteria form a dynamic ecosystem that acts to maintain the health of the neonate (e.g., stress responses, neuronal growth, plasticity, and neurotransmission) and that modulate the neuro-immune axis [151,157]. However, preterm born and FGR infants are reported to have an altered microbiota, characterized by delayed colonization of beneficial bacteria, reduced diversity of organisms involved in the development/maintenance of a healthy gut microbiome [158,159], and increased populations of potential pathogenic microorganisms [160]. The impact of this gut dysbiosis on brain development and the interactions between perinatal inflammation and gut–brain axis function remains unclear. Nevertheless, multiple studies have suggested a role for an abnormal microbiome in autism spectrum and attention deficit hyperactivity disorders [161,162,163], both of which are associated with PE. Early alterations to the gut microbiota can also have long lasting effects on immune activation [164,165]. For example, preterm born infants have higher levels of facultative anaerobic microorganisms and reduced levels of strict anaerobes in their microbiome [160], which may contribute to delayed maturation of the immune system and increased risk of perinatal infection. Interestingly, in germ-free mice, the complete absence of gut microbiota is associated with impaired development of brain microglia, resulting in impaired innate immune responses [166].

## 3. Key Cellular and Molecular Players in Neuroinflammation

From an allostasis perspective, “…acute stress enhances immune function whereas chronic stress suppresses it, [which] can be beneficial for some types of immune responses and deleterious for others” [167]. Under this framework, the mechanisms that alter developing brain structure or function might reflect the consequences of inflammation resolution [168] or repair [169], or most likely a mix of both [170,171,172]. Indeed, as detailed below, classical mediators of inflammation such as microglia, astrocytes, cytokines, and chemokines can have both beneficial and deleterious consequences on brain development and function. Under homeostatic conditions, these inflammatory mediators play important roles in normal brain development and function, including synaptogenesis and circuit refinement [173,174,175], apoptosis [176,177], angiogenesis [178], and progenitor cell maintenance, proliferation, differentiation, and migration [179,180]. However, in the preterm neonate, systemic infection/inflammation or sterile inflammation (i.e., that secondary to mechanical ventilation and other events) can invoke a pathophysiological inflammatory response in the brain, including microglial activation and an imbalance in the production of chemokines/cytokines, which can trigger and/or exacerbate brain injury [181,182,183,184].

### 3.1. Chemokines and Cytokines

Chemokines are small polypeptides secreted by activated immune cells, as well as non-immunes cells in the CNS such as neurons, astrocytes, and oligodendrocyte progenitor cells. They function as chemoattractant cytokines and are important for the migration of inflammatory cells (e.g., immune cells and endothelial cells) from the vascular system to the site of inflammation [185,186]. Cytokines are small molecular weight proteins capable of diffusing over the BBB, and are involved in crosstalk between the CNS, endocrine system, and immune system to maintain CNS homeostasis. Cytokines can be pro- or anti-inflammatory, and in response to injury, the pro- and anti-inflammatory cytokine balance can determine whether the inflammatory response is neuroprotective [187,188] or damaging [189,190,191,192].

As previously discussed, the inflammatory response to systemic immune stimulation can promote the entry of pathogens and peripheral immune molecules (e.g., cytokines, leukocytes, and adhesion molecules) into the brain [193,194]. This, in turn, can trigger a neuroinflammatory response involving activation of CNS microglia and secondary release of local cytokines and other cytotoxic mediators (e.g., reactive oxygen and nitrogen species, and associated free radicals) [195,196]. This can directly damage the developing brain by impairing cellular mitochondrial function [189], inducing glutamate toxicity [197] and cellular apoptosis [190,198], and disrupting the proliferation and differentiation of neurons and oligodendrocytes [192,199,200,201]. Pro-inflammatory cytokines are also proposed to injure the developing brain by enhancing CNS production of prostaglandin E2 via cyclooxygenase-2 activation [202,203].

Numerous clinical and experimental studies have shown associations of elevated systemic and CNS chemokines/cytokines with adverse neurological outcomes. Preterm born infants with inflammation-related brain injury show higher concentrations of pro-inflammatory cytokines (e.g., IL-1β, IL-6, and TNF-α) and chemokines (e.g., IL-8 and monocyte chemoattractant protein-1) in the amniotic fluid [204], umbilical cord blood [182,205,206], cerebrospinal fluid [207], and brain tissues [208] compared with healthy term infants. In very preterm born babies, increased TNF-α levels in the umbilical cord blood was associated with depressed brain electrical activity within the first 72 h of life [209] and impaired cognitive function at 2 years of adjusted life [209,210]. Increased concentrations of TNF-α, IL-1β, IL-6, and IL-10 in umbilical cord blood of very preterm born infants were also predictive of cerebral lesions detected by MRI following intrauterine inflammation [211]. Further, human post-mortem studies have shown microglial activation and upregulation of pro-inflammatory cytokines in the brains of preterm infants with evidence of white matter injury [198,212,213].

### 3.2. Microglia

Microglia are the resident immune cells of the CNS, providing the first line of defense against invading microorganisms, preventing collateral inflammation-induced damage, and protecting the brain during injury and disease [214]. However, following injury, activated microglia can exhibit both protective and damaging roles in the brain. Under normal physiological conditions, microglia acquire a ramified morphology, playing a key role in maintaining brain homeostasis [215], neuronal survival [177,216], synaptic pruning, and regulating large-scale connectivity [173]. However, in response to infection/inflammation [217,218,219] or sterile inflammatory responses (e.g., after hypoxia-ischemia) [212,213,220,221], activated microglia rapidly become amoeboid, and can lose their normal developmental and homeostatic functions, becoming detrimental to the immature brain [218,219]. For example, microglial activation can cause secondary release of locally-produced pro-inflammatory cytokines such as TNF-α and IL-1β [219], which can promote further microglial activation and cause direct cell injury. This, in turn, can further increase BBB permeability and promote further entry of macrophages and leukocytes into the brain [193,194]. Uncontrolled, sustained activation of microglia can also cause excessive release of excitotoxic molecules, such as glutamate, which can directly induce cell death [196,222,223]. Further, microglial activation can produce reactive oxygen and nitrogen species, which induce cellular damage via various signaling pathways [218,224,225]. Microglia have been described as a possible link between maternal immune activation, PE, and disturbed neurodevelopment [226].

### 3.3. Astrocytes

Under normal conditions, astrocytes play key roles in maintaining CNS homeostasis and neural function by providing energy and structural support, glucose metabolism, removal of waste, and regulating neuronal proliferation, maturation, survival, and signaling [227]. Importantly, astrocytes are also thought to modulate CNS innate immune responses and express a wide range of bacterial and viral pathogen receptors. For example, under normal conditions, astrocytes promote an anti-inflammatory environment via the production of transforming growth factor (TGF)-β [228] and regulate the trafficking of lymphocytes across the brain endothelial barriers [229]. However, following infection/inflammation or secondary to hypoxia-ischemia, astrocytes can contribute to the pathogenesis of both white matter and grey matter injury [230,231]. For example, activated astrocytes can release a range of cytotoxic molecules (e.g., inducible nitric oxide synthase) and cytokines [191,232]. In vitro studies have shown that in response to pro-inflammatory cytokines, white matter astrocytes can also trigger arrest of pre-oligodendrocyte maturation via induction of cyclooxygenase-2-prostaglandin E2 signaling [202], or by increased production of astrocyte-derived platelet-derived growth factor [233]. Reactive astrocytes can also inhibit oligodendrocyte maturation and remyelination by increasing Notch signaling in rodent models of multiple sclerosis [234], although the significance in preterm brain injury is unclear. Further, following immune stimulation in neonatal animal models, astrocyte activation was associated with impaired dendritic complexity and spine formation, impaired cognition function in later life [235], and decreased pre-synaptic vesicle density [236].

## 4. Frequently Used Animal Models of Neuroinflammation

The development of in utero and postnatal animal models is critical for establishing a causal relationship between early-life immune exposures and neurodevelopmental outcomes and for identifying cellular and molecular mechanisms that alter normal brain development. The most frequently used designs focus on bacterial/viral, cytokine, or FGR models of inflammation, but most are unable to separate the influences of preterm delivery from the consequences of PE.

### 4.1. Bacterial and Viral Models of Inflammation

A variety of agents are used to mimic the inflammatory response induced by bacterial and viral infections, in the absence of live bacteria or virus exposure. Lipopolysaccharide (LPS) is a cell wall component of the gram-negative bacteria Escherichia coli, and activates inflammatory responses via binding to TLR4 [237], while polyinosinic:polycytidylic acid (poly IC) is a synthetic analogue of double stranded RNA generated during viral infections, and activates inflammatory responses via binding to TLR3 [238,239,240]. Maternal/fetal exposure to poly:IC or LPS has been used to model the effect of prenatal inflammation on neonatal outcomes. For example, high dose poly:IC (10 mg/kg) to pregnant mice resulted in spontaneous abortion, whereas lower doses of poly:IC (2.5 mg/kg and 5 mg/kg) resulted in dose-dependent immune system activation [241]. Lower doses of poly:IC to pregnant mice at mid-gestation acutely also decreased brain IL-10 concentrations, increased IL-6 concentrations, and suppressed spatial exploration in juvenile rats [242]. Interestingly, administration of the same dose of poly:IC in late gestation caused an acute increase in hippocampal apoptosis, and in brain IL-1β and IL-10 concentrations, as well as persisting behavioral deficits [242].

Administration of low dose LPS (0.1 mg/kg) to pregnant rats at mid-gestation was associated with reduced litter size and reduced social interactions in both juvenile and adult offspring [243]. By contrast, low dose LPS (0.5 mg/kg) in pregnant rats in late gestation increased fetal brain cytokine mRNA expression (IL-1β, TNF-α), increased gliosis in the hippocampus, and reduced myelin basic protein staining [192]. Further, intrauterine administration of low dose (125 μg/dam) or high dose (250 μg/dam) LPS at mid gestation caused fetal death [244], while low dose LPS at late gestation was associated with decreased brain weights, hypomyelination, enlarged ventricles, and cortical grey matter lesions [245]. Additionally, intravenous single bolus (0.1–0.2 mg/kg) or repeated (1 µg/kg) low dose LPS to preterm fetal sheep caused both diffuse and cystic white matter damage and inflammation [217,246,247]. Further, chronic low-dose exposure to LPS (100–250 ng) for 5 days with superimposed 1 μg boluses was associated with white matter inflammation and loss of mature oligodendrocytes [248,249]. Although there is less experimental evidence for a direct effect of prenatal infection/inflammation on grey matter development, in pregnant rabbits, intrauterine administration of LPS during late gestation was associated with decreased dendritic arborization and spine density in the retrosplenial cortex of newborn kits [250]. Further, in utero exposure to single bolus or continuous low dose LPS in preterm fetal sheep was associated with neuronal cell death and loss of the normal maturational increase in cortical EEG amplitude [246,251].

Postnatal administration of poly IC induces a similar spectrum of impaired behavioral outcomes as prenatal exposure, which range from impaired social interactions to memory impairments. For example, repeated postnatal administration of low dose poly:IC (5 mg/kg) impaired cognitive function and glutamatergic neurotransmission, without evidence of gross brain pathology, in young adult rats [235]. Further, single postnatal administration of LPS systemically [252] or into the brain [253] induced significant upregulation of central pro-inflammatory cytokines (e.g., TNF-α, IL-1β, and IL-6), reactive gliosis, and white matter hypomyelination in newborn rodents [254]. Similarly, single or repeated postnatal systemic administration of LPS in newborn rodents was associated with CNS inflammation and impaired brain development, including impaired oligodendrocyte maturation, reduced white matter myelination, decreased grey matter volumes, impaired dendritogenesis of cortical neurons, and reduced numbers of hippocampal synapses [255,256,257,258]. Neuroinflammation also appears capable of eliciting endothelial dysfunction [259], a hallmark of PE.

### 4.2. Cytokine Models of Inflammation: IL-1β

In mice, systemic administration of IL-1β during the first 5 postnatal days (a stage of brain development that broadly corresponds to the first half of the third trimester of pregnancy in humans) was shown to mimic several key features of the encephalopathy of prematurity, including impaired memory and social behavior, altered white matter microstructure, microglial activation (pro-inflammatory phenotype), delayed myelination linked to delayed maturation of oligodendrocytes, reduced density of selective subsets of interneurons, and abnormal synaptic equipment [199,260,261,262]. Interestingly, males are more affected than females. Using this model, it was also demonstrated that microglial activation was responsible for the oligodendrocyte maturation blockade, likely through a cross talk with astrocytes [262,263]. Mechanistic studies of microglia activation have shown a key role of microglial Wnt pathway inhibition and of microglial PSD95 [261,262].

### 4.3. FGR Models of Inflammation

Experimentally, brain abnormalities observed in models of FGR have been related to excitotoxicity, oxidative stress, cell death, and neuroinflammation with microglial activation and reactive astrogliosis [264,265,266,267,268]. Neuroinflammation, and in particular microglial activation, can contribute to oligodendrocyte maturation blockade [199,262]. In a model of FGR based on protein restriction during pregnancy, transcriptomic analysis identified multiple alterations in the expression of genes involved in inflammatory pathways, both in microglia and oligodendrocytes [269].

## 5. Clinical Indicators of Damage or Dysmaturation to Developing Brain Structure

### 5.1. Neuronal Migration Disorders

Disruption of normal developmental processes in early pregnancy can result in holoprosencephaly, microcephaly, and neuronal migration disorders that appear to underlie one in ten cases of cerebral palsy [270], a condition that has been associated with PE, FGR, and PTD. At least one large case–control study reported that PE is associated with microcephaly in offspring, while gestational hypertension is not [271]. Interestingly, these disorders are often identified in pregnancies that reach term.

### 5.2. White Matter Damage and Dysconnectivity

White matter damage is the major pattern of brain injury observed in preterm infants. In historical cohorts, this was characterized by severe necrosis (e.g., periventricular leukomalacia), with oligodendrocyte cell death, axonal degeneration, and loss of myelin [272]. On the heels of vast improvements in neonatal care, the incidence of this severe white matter injury is now low in modern preterm cohorts (<5% of cases) [273,274,275,276,277,278]. However, this has been replaced by a less severe (non-necrotic), but more diffuse deficit in white matter myelination and reduced white matter volumes [278,279,280,281,282,283,284,285,286], which apparently manifests as milder forms of neurobehavioral and intellectual disabilities [287,288,289,290,291,292,293,294,295]. Indeed, MRI studies have repeatedly confirmed that by term-equivalent, preterm infants have reduced cortical and subcortical (e.g., striatal and thalamic) volumes, without evidence of gross pathology, compared with term born controls [296,297]. Moreover, reduced brain volumes persist into adulthood and are associated with cognitive deficits [296].

Diffuse white matter injury is characterized by acute degeneration of pre-myelinating oligodendrocytes (preOLs) and reactive astrogliosis [272,298,299,300], proliferative regeneration of the preOL pool, and chronic failure of oligodendrocyte maturation [199,213,258,301,302,303,304,305,306,307,308]. A similar failure of oligodendrocyte maturation has been reported in cases of less severe white matter injury without evidence of acute oligodendrocyte cell death [199,302]. In either case, the chronic deficits in white matter myelination in modern cohorts of preterm infants are now considered to represent a failure of oligodendrocyte maturation [309,310,311,312]

Interestingly, PE has been associated with reduced risk of neonatal white matter injury compared with other contributors to very preterm birth in some studies [5,313]. However, combinations of PE and FGR with preterm birth and related sequelae (i.e., postnatal inflammation) supports the concept of multiple-hit models, where postnatal insults contribute to increased risk of white matter injury among children born very preterm [314,315]. FGR is also associated with widespread changes in white matter maturation at school age among children born very preterm [316]. Further, in a more recent study of FGR children, those who had adverse outcomes exhibited microstructural changes in the frontal white matter by MRI (i.e., a reduced apparent diffusion coefficient) more often than their FGR peers who did not develop adverse outcomes [317]. Importantly, white matter injury also occurs among children born at term; e.g., a pilot MRI study reported evidence of altered white matter development in children whose mothers had PE compared with controls also born at term [318].

### 5.3. Grey Matter Injury

Imaging studies also show that preterm birth is associated with long-term reductions in growth and connectivity of cortical and subcortical grey matter structures (e.g., striatum, thalamus, hippocampus) [276,278,319,320,321,322,323,324,325,326], decreased cortical surface area, complexity, and folding [325,327,328], and delayed gyral maturation [296,329]. These deficits in grey matter development are highly associated with adverse neurodevelopmental outcomes [326,330,331,332], including impaired cognition and lower IQ [294,296,325,333,334], impaired memory and learning ability [291,335,336], and impaired executive function [337,338,339]. At least one study has also reported grey matter deficits in FGR children born very preterm, including in the amygdala, basal ganglia, bilateral thalamus and insula, left occipital and parietal lobes, and right perirolandic area [340]; in that study, affected children also had increased fractional anisotropy in the forceps minor, internal and external capsules, uncinated, and fronto-occipital white matter tracts, suggestive of altered white matter development.

In historical cohorts of preterm infants with severe cystic white matter injury, these grey matter changes likely relate to widespread neuronal cell death [341,342,343,344,345]. However, post-mortem studies of preterm infants with diffuse white matter injury have found limited evidence of acute neuronal degeneration or overt grey matter injury [213,346]. As an alternative mechanism, recent experimental animal studies reported reduced growth and complexity of neuronal dendrites and synapses in cortical and subcortical grey matter structures following preterm birth [347], and after neonatal hypoxia-ischemia [331,348,349,350] and systemic inflammation [250,258,260]. In both preterm fetal sheep and newborn rats, reduced neuronal complexity was associated with loss of the developmental decline in fractional anisotropy [258,331,349,351]. Preterm infants were also reported to have higher cortical fractional anisotropy values at term than normal term born infants, which was strongly associated with impaired cortical growth and worse neurodevelopmental function at 2 years of age [326,352]. Further, in a small case series, some preterm born infants showed reduced dendritic complexity and numbers of neuronal spines of cortical neurons [353], consistent with arrested neuronal development.

## 6. Key Mechanisms

### 6.1. Infection

Intrauterine infection is thought to occur in 25% to 40% of all preterm births, although molecular and histological examinations suggest that this is an underestimate [354,355,356,357,358]. Human studies consistently show that brain injury and disability in preterm infants are highly associated with exposure to both severe/repeated and mild (including subclinical) infections and inflammation during fetal and early postnatal life [359,360,361]. Evidence of intrauterine infection such as acute-chorioamnionitis [210,362] is associated with white matter injury [359,363,364]. I, increased risk of cerebral palsy [365], poor neurodevelopmental outcomes [366,367,368,369], and overall disability [370].

The majority of very preterm infants also develop at least one postnatal infection while in intensive care [99,363,371,372,373,374,375]. Severe postnatal infections such as culture-positive sepsis, meningitis, or necrotizing enterocolitis are associated with white matter injury [374,376,377], long-term impairment of learning/memory, visual, and auditory function, and cerebral palsy [378,379,380,381,382,383,384]. Multiple infections/inflammatory events are associated with increased risks [65,381,385,386,387]. Further, more mild but continued postnatal systemic inflammation in preterm infants is associated with both white matter and grey matter injury [325,387,388,389,390,391] and a range of poor neurological outcomes [99,359,392,393]. Importantly, infection even before pregnancy can convey information about increased risks of pregnancy and postnatal complications (e.g., cervical-vaginal infections [66,67]).

### 6.2. Hypoxia–Ischemia and Immune Activation

The prevalence of acute asphyxia at birth with a base deficit >12 mmol/L and early onset hypoxic-ischemic encephalopathy occurs much more frequently in preterm infants and is strongly associated with adverse outcomes [394,395]. On the other hand, the relevance of hypoxia-ischemia in observational studies of neonatal encephalopathy remains unclear; first, because we cannot directly measure hypoxia-ischemia, but also because the majority of children who are exposed to clinical indicators of asphyxia are free of major handicap at school age (see [396] for a review). Importantly, the grammar used by basic scientists often refers to oxygen deprivation long before labor (e.g., in the first trimester) that is not intended to generalize to intrapartum asphyxia per se.

Milder hypoxia-ischemia in preterm fetal sheep is associated with both white matter and gray matter dysmaturation, as shown by reduced arborization of cortical and striatal neurons without evidence of overt neuronal loss [330,348] and acute death of pre-oligodendrocytes, regenerative proliferation, and later dysmaturation [303]. Although it is difficult to precisely ascribe the etiology, preterm fetal sheep studies provided evidence that hypoxia-ischemia upregulates circulating pro- and anti-inflammatory cytokines and induces chronic microglial infiltration and astrogliosis between 3 and 21 days after the insult [96,397]. Similarly, in preterm and term infants, neonatal encephalopathy is highly associated with increased central and peripheral cytokine levels [398,399,400]. Indeed, extensive evidence supports the possibility that chronic upregulation of systemic and CNS cytokines (e.g., systemic upregulation of TNF-α and IL-1β) and secondary gliosis are associated with heightened risks of neurodevelopmental problems after preterm birth [209,401]. Thus, secondary inflammation, a process that is measurable in human observational studies, might augment or mediate the brain damage and dysmaturation that are reported in experimental studies of hypoxia-ischemia [402]. Oxidative stress in the placenta might play key roles as well [403,404,405].

## 7. Future Directions to Identify Viable Therapeutic Targets

### 7.1. Preconception and Early Pregnancy Interventions

PE is diagnosed after the 20th week of gestation by definition, but the underlying blood pressure irregularities and indicators of chronic systemic inflammation (and their antecedents [406]) are often detectable much earlier. This is the premise for administering LDA in the first trimester for the prevention of PE in some high-risk women. 

Animal models strongly support the possibility of therapeutic intervention in early pregnancy for the prevention of neurodevelopmental disorders, because multiple environmental exposures can impair neuronal (and other cellular) migration and maturation in what translates to the first and second trimesters of human pregnancy [407]. Moreover, observational studies have shown an overrepresentation of brain malformations and neuronal migration disorders, but also cardiovascular birth defects, in the offspring of mothers with PE [3]. Indeed, early onset PE appears particularly preferentially associated with major fetal cardiac defects [408,409,410,411], which has prompted discussion of the potential for a common cause [410,412,413]. Interestingly, at least one population-based study has provided evidence that maternal congenital heart disease is associated with increased risk of PE [414].

Recent evidence from a preclinical study of human and chicken embryos also implicates chronic fetal hypoxia and FGR in the pathophysiology of cardiovascular dysfunction, prompting hope that antenatal intervention might even disrupt what appear to be the fetal origins of adult chronic disease [415]. The Rotterdam study inspires much more enthusiasm by providing observational evidence that embryonic growth is clinically measurable, associated with paternal and maternal risk factors (e.g., alcohol consumption [416]), and appears to be amendable to clinical intervention [417]; moreover, the investigators reported smaller total grey matter and cortical volume in children whose mothers exhibited thyrotropin irregularities during the first trimester [418] (a correlate of PE that is also associated with poor air quality [419], itself a risk factor for PE and eclampsia [420]), and more recently they developed growth trajectories for the embryonic head that might be useful for identifying early antenatal abnormalities [421].

### 7.2. Second or Third Trimester and Postnatal Interventions

The Extremely Low Gestational Age Newborn (ELGAN) Study of children born prior to the 28th week of gestation offers compelling observational evidence that sustained or intermittent postnatal systemic inflammation during the first few postnatal weeks conveys information about elevated risks of neurobehavioral problems among survivors [422]. FGR that was mostly combined with PE was identified as a key risk factor for sustained postnatal inflammation [423,424], but also for autism and non-autistic social impairment. Similar phenomena might occur in utero among pregnancies that carry into the third trimester (i.e., the premise of the ELGAN Study). Importantly, the ELGAN Study provides compelling evidence that neurotrophins might modify the association between sustained systemic inflammation and adverse neurobehavioral developmental outcomes, hinting at therapeutic potential. Experimental studies support this possibility [425,426,427], as does observational evidence [426].

### 7.3. Sources of Complexity

Preconception interventions might be effective as early as mid-adolescence to prevent hypertensive disorders of pregnancy (and downstream sequelae including FGR) [406]. For example, China’s National Maternal Near Miss Surveillance System recently reported that two-thirds of an observed decrease in children born small for gestational age from 2012–2020 could be accounted for by changes in maternal characteristics (e.g., education level) [428]. Therapeutic intervention studies will likely benefit from incorporating information about low-grade systemic inflammation at periconceptual enrollment, based on indirect evidence that obesity (or its correlates) might mitigate the effectiveness of LDA for the prevention of miscarriage [69,429].

There is ample evidence to suggest that antenatal intervention can improve neurobehavioral developmental outcomes in children whose mothers have PE or possibly other inflammation-invoking conditions during pregnancy. For example, antenatal magnesium sulfate administered to prevent seizures in preeclamptic women and prolong their preterm pregnancies for up to two days is also associated with a significantly reduced risk of cerebral palsy in offspring [430], although the benefits appear more difficult to detect among school-aged children [431].

Postnatal therapeutic intervention is likewise plausible based the effectiveness of exogenous thyroxine for improving neurodevelopmental outcomes in children with low circulating thyroid hormone levels; however, expanded use-cases beyond congenital hypothyroidism have proven difficult to materialize (e.g., transient hypothyroxinemia of prematurity [432]). More concerning is recent evidence that hints at potential harms from postnatal administration of exogenous erythropoietin for fetal neuroprotection in high-risk newborns [433]. Indeed, evidence mostly from animal models raises serious concerns about the use of antenatal steroids for enhancing fetal lung maturation [434] or even antenatal magnesium sulfate for neuroprotection [435].

We do not know why exogenous thyroxine prevents neurodevelopmental problems in some children but not others. Much less is known about the potential benefits or harms from exogenous erythropoietin for neuroprotection, namely because human trials were conducted without establishing the significance of endogenous erythropoietin in human neurodevelopment. Much like thyroxine, erythropoietin boasts a plethora of pleiotropic properties beyond what’s involved in the maintenance of optimal tissue oxygenation. For example, pre-clinical and clinical evidence is consistent with anti-inflammatory and growth/trophic properties, but endogenous erythropoietin might also act as a tissue protector or it is equally likely that it reflects immaturity/vulnerability of the brain or of the systems responsible for protecting it [436]. Moreover, the ELGAN study showed that heightened endogenous erythropoietin by itself conveyed information about very low mental and physical development scores and microcephaly, but when combined with heightened intermittent or sustained systemic inflammation it was further associated elevated risks of ventriculomegaly and hemiparetic cerebral palsy [437].

Even more complexity is introduced by the associations between indicators of social vulnerability, systemic inflammation-related phenomena, and hypertension (before, during, and after pregnancy). The relationship between social vulnerability and systemic inflammation has been replicated internationally across multiple contexts [438]. Multiple indicators of social disadvantage and inflammation-related phenomena are associated with increased risks of preterm birth [439,440,441] and potentially related sequelae in offspring (e.g., neurobehavioral deficits [442,443,444,445,446,447,448,449,450] and academic underachievement [449]), but the mechanisms are poorly understood (i.e., insights have not produced effective interventions) [451]. While maternal stress [452,453], pre-pregnancy obesity [454,455,456], gestational weight gain [457], and indicators of maternal immune-activation [438,458,459,460,461,462] and related factors (i.e., hypertension) play key roles, the pathways to adverse pregnancy and developmental outcomes appear mediated by the developing placenta’s stress response [463].

The earliest deliveries appear to involve acute inflammatory responses in the placenta more so than chronic inflammation. For example, US black women are much more likely than white women to suffer periviable gestational lengths [464], and severe histologic acute chorioamnionitis is fourfold more common in black than white mothers who deliver very preterm [465]. On the other hand, chronic hypertension [466] and histologic evidence of abnormal or incomplete trophoblast remodeling of maternal spiral arteries are more prevalent in US black than white women, and exposure is associated with increased risk of later preterm delivery [467]. Importantly, multiple indicators of social vulnerability are associated with increased vascular resistance [468] and chronic inflammation [461] in the placenta. The distinction between acute and chronic inflammation might be somewhat artificial or oversimplified in the real world, because the earlier the preterm delivery, the greater the overlap between PE, FGR, placental malperfusion, and both acute and chronic placental inflammation, plus the higher the risks of neurobehavioral sequelae in offspring [469,470,471,472,473]. Indeed, both antenatal and postnatal systemic inflammation are associated with increased risks of indicators of damage to brain structure or function among children born very preterm [386], perhaps acting akin to a double (or multi) hit model of pathogenesis [314]. Importantly, the association appears to be dose-related [474]; i.e., the worst pregnancy outcomes are seen with combinations of antenatal and postnatal inflammation [386] or with acute and chronic placental inflammation [65].

Recent studies of basic income provide invaluable insights about the possibility of social interventions to improve neurodevelopmental outcomes. For example, at least one randomized control trial showed that a predictable, monthly unconditional cash transfer given to low-income families may have a causal impact on infant brain activity [475]. The other side of this coin is that failure to incorporate information about dynamic social stressors in human observational and experimental studies might raise the risks of false negative and false positive errors.

## 8. Conclusions

The evidence consistent with convergence to a final common pathway to neurodevelopmental problems involving arrested brain maturation and dysconnectivity raises an intriguing possibility that chronic systemic inflammation hints at viable therapeutic targets in the context of multiple pregnancy disorders, both in early gestation and beyond. Carefully designed hypothesis-driven studies, however, are likely needed to translate the mechanistic evidence into population health benefits, namely because inflammation-related phenomena occurring before, during, and after pregnancy can each convey dynamic information about heightened risks of neurodevelopmental problems in offspring. Moreover, chronic inflammation is associated with increased systolic blood pressure, and both conditions are overrepresented in socially vulnerable populations. In summation, exposure to chronic systemic inflammation-related phenomena appears to start early on and might be additive in conveying information about neurodevelopmental problems in offspring. This prompts us to infer that effective therapies to alleviate chronic inflammation might improve maternal and offspring outcomes alike.
